# Subjective and Objective Day-to-Day Performance Measures of People with Essential Tremor

**DOI:** 10.3390/s24154854

**Published:** 2024-07-26

**Authors:** Navit Roth, Adham Salih, Sara Rosenblum

**Affiliations:** 1 The Laboratory of Complex Human Activity and Participation (CHAP), Department of Occupational Therapy, University of Haifa, Haifa 3498838, Israel; navroth@braude.ac.il; 2 Department of Mechanical Engineering, Braude College of Engineering, Karmiel 2161002, Israel; adhamsalih@braude.ac.il

**Keywords:** essential tremor, functional disability, ADL, modifications

## Abstract

This paper aims to map the daily functional characteristics of people diagnosed with essential tremor (ET) based on their subjective self-reports. In addition, we provide objective measurements of a cup-drinking task. This study involved 20 participants diagnosed with ET who completed the Columbia University Assessment of Disability in Essential Tremor (CADET) questionnaire that included five additional tasks related to digital equipment operation we wrote. Participants also described task-performance modifications they implemented. To create objective personal performance profiles, they performed a cup-drinking task while being monitored using a sensor measurement system. The CADET’s subjective self-report results indicate that the most prevalent tasks participants reported as having difficulty with or requiring modifications were writing, threading a needle, carrying a cup, using a spoon, pouring, and taking a photo or video on a mobile phone. Analysis of participants’ modifications revealed that holding the object with two hands or with one hand supporting the other were the most prevalent types. No significant correlation was found between the CADET total scores and the cup drinking objective measures. Capturing patients’ perspectives on their functional disability, alongside objective performance measures, is envisioned to contribute to the development of custom-tailored interventions aligned with individual profiles, i.e., patient-based/smart healthcare.

## 1. Introduction

Essential tremor (ET), one of the most common movement disorders, is found in a wide age range, with a prevalence of about 5% in people over 65 years [1]. The disorder is characterized mainly as an action tremor (postural or kinetic) but can also appear while at rest [2,3,4]. The frequency of the tremor in ET may range between 4 and 12 Hz and decreases with time [5]. Although ET may affect assorted body parts, most often upper limbs are affected with a slight asymmetry [6,7]. Most people with ET do not seek medical diagnosis [1,6,8,9]. About 85% report that the tremor causes functional disability [10] in the activities of daily living (ADL)—such as eating, drinking, and using tools and self-care instruments that require fine and gross motor skills [10] and in work performance [6].

Previous studies indicated that stress and fatigue may also affect the tremor, whereas non-motoric symptoms, including changes in cognition, depression, and anxiety, may also be involved among patients with ET [6,9,11,12]. The potential for ET to cause embarrassment and social consequences [6] seems to influence the patients’ decisions to seek medical treatment [13]. Embarrassment exists in almost 50% of those with mild ET (even without head tremor) [13], and the social effects may result in patients avoiding social meetings and tasks that will expose the tremor. These studies implied that ET and its associated depression and anxiety may cause a decline in quality of life among people with ET [14,15,16]. 

Currently, ET is clinically diagnosed using a combination of a neurological examination and a medical history review [17]. Assessment of tremor severity may include scales that are not ET-specific, such as the Fahn-Tolosa-Marin Clinical Tremor Rating Scale [18] and scales developed specifically for ET such as the Tremor Research Group Essential Tremor Rating Assessment Scale [19]. 

Usually, functional disability due to tremors is assessed with self-report questionnaires and performance-based tests [20] that may be part of a severity-assessment scale. Self-report questionnaires provide assessors with the perspective of the person coping with the tremor by asking respondents whether they have difficulty or disability in ADL tasks and how difficult those tasks are to perform. Such instruments are the Bain and Findley ADL scale [21] and the Columbia University Assessment of Disability in Essential Tremor (CADET) questionnaire [22]. The CADET, which was implemented in the current study, is a valid and reliable 31-item self-report questionnaire. It focuses on the respondent’s difficulty/disability while performing ADL tasks and whether the respondent made modifications (or had a change in efficiency) [22,23]. Eliciting this information is especially valuable because people who make modifications to perform ADL do not necessarily report having a disability [24]. Modifications include changing the environmental, psychosocial, behavioral, psychological, or physical method of performing a specific task [25]. 

In previous studies, the highest percentage of people with ET who were surveyed reported the physical tasks of using a spoon, drinking from, holding, or carrying a cup, pouring a drink, and of writing were difficult to perform, required modification, or carried out less efficiently than previously [6,10]. To the best of our knowledge, however, there is no detailed documentation on the modification types/profiles they implemented in various daily tasks. Thus, this current study focuses on physical modifications. In addition, because we did not find detailed documentation on the reported disability while performing diverse tasks of digital and smartphone operation, we made these tasks a secondary focus of our research.

Another aim of this paper is to demonstrate the need for performance-based tests that supply objective measures of day-to-day performance. Measurements—to quantify tremors in various ADL tasks—may be acquired using digital-graphic boards, accelerometers and gyroscopes, image processing, and electromyography [20,26,27,28]. The resulting metrics may be implemented as reciprocal validation [29] of neurology assessments and/or self-report scales [20], providing support for the individual’s functional health status evaluation. 

In this study, the performance-based objective measures were gathered for the cup-drinking task as an example of a functional daily task that challenges people with ET [6,21,22]. This particular task was chosen as more than 65% of participants with ET reported difficulties/disabilities or needed modification/efficiency changes while performing the task [10,27]. In addition, this daily task comprises multiple phases with different kinematic features [30], during which tremor characteristics, which may differ with posture and force levels, may affect tremor amplitude [31,32]. The cup-drinking task was used by the Glass scale [33] as one task for assessing tremor severity among people with ET.

In previous studies, objective measures of the tremor were obtained while performing cup tasks using hand or cup-attached accelerometers in drinking and holding-a-cup tasks [26,34,35] or by measuring spilled water volume in the holding-a-cup task [27]. To the best of our knowledge, however, the correlation between the objective drinking task measures and the total score of a self-report disability questionnaire was not reported and thus was examined in the current study. 

In line with the personal medicine view [36], understanding patients’ perspectives and the ways they perform daily tasks may aid in the pre-clinical phase, as well as in assessment and treatment processes, toward improved physical and emotional health and quality of life [25]. Thus, this study also aims to map activity performance features of people who are diagnosed with ET but who may not be followed routinely or medically. To this end, we combine the individual patient’s responses on a self-report disability questionnaire and objective performance-based quantitative measures for the common ADL task of drinking to create an individual profile.

## 2. Methods

### 2.1. Participants

We recruited participants by advertising among the general population (e.g., via social media, bulletin boards, and senior culture clubs). Inclusion criteria were being aged 20 or more and having a written physician’s diagnosis of ET. Exclusion criteria were another disability that could affect the participants’ tremors based on their self-reports (previously also described) [37]. Twenty people (N=20) met the inclusion and exclusion criteria and participated in the study. This study was conducted according to the guidelines of the Declaration of Helsinki; the University of Haifa Ethics Committee approved the study (018/19). All participants signed informed consent forms.

### 2.2. Instruments

Participants who met the criteria signed an informed consent form and completed a demographic questionnaire, a Mini-Mental State Examination [38], and an expanded and translated CADET questionnaire, including questions on extra digital tasks [22]. In addition, they performed an ADL task of drinking from a cup using three grip types. 

#### 2.2.1. Demographic Questionnaire

The demographic questionnaire included information on the participants’ age, education (years), dominant hand, tremor-specific medication, general medication, duration (years) since noticing the tremor, duration (years) since diagnosis with ET, and family members diagnosed with ET.

#### 2.2.2. CADET Questionnaire with Further Questions on Extra Tasks and Modifications

To address disability in daily tasks due to the tremor, a Hebrew translation of the validated CADET questionnaire was administrated after approval by the corresponding author [23]. The 31 tasks were rated as 0 (no disability, need to modify activities, or no loss of efficiency), 1 (no disability but a need to modify activities or a loss of efficiency), or 2 (disability or both a need to modify and loss of efficiency) [10].

Questions related to the five digital-equipment operation tasks were added for this study: (1) connect to digital accessories (e.g., connect a phone charger, headphones, and a USB drive); (2) select an object with a computer mouse (e.g., select and click on an object from a toolbar or a recipient from an e-mail list); (3) type digits or a text message with fingers on a mobile phone; (4) take a photo or video using a mobile phone; and (5) use a finger to choose an object on a mobile phone screen (e.g., an application icon). The CADET outcome measures comprised two mean scores: one for the questionnaire’s original 31 items and the other for the five questions related to the extra tasks. Participants also listed up to five tasks in which the tremor bothered them the most and elaborated on modifications in the task performance they made due to the tremor. 

#### 2.2.3. The Cup Drinking Task: Objective Performance-Based Measures 

As described in the introduction section, the ADL task of drinking has been chosen as a representative ADL task for the objective measurements. This task included lifting a cup from the table, moving the cup toward the mouth, performing a drinking-like movement, and returning the cup to the table. Participants were seated in front of a table with the cup positioned on it at about 25 cm from the edge in front of the participant’s dominant hand. This layout was applied to enable the task simulation of picking up the cup and drinking in their regular manner during a meal. Participants were instructed to perform the task in their regular manner as close as possible to their real-life daily performance but using three grip types: (i) around-the-cup grip (without using the handle), (ii) below-the-handle grip (leaning the handle on the fingers), and (iii) on-the-handle grip (holding only the handle).

**Figure 1:**
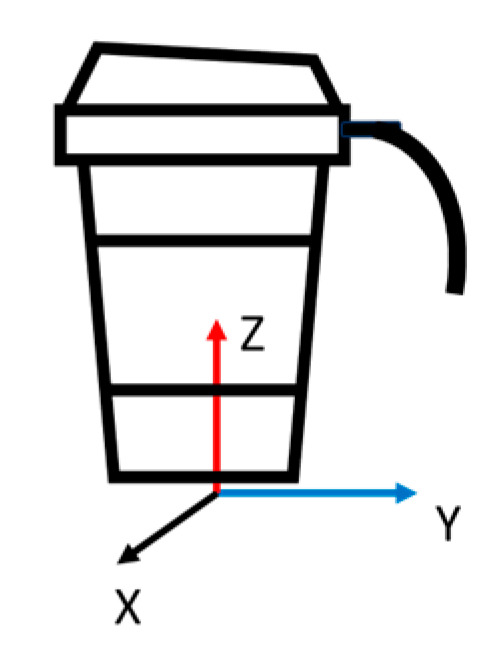
Cup accelerometer axes layout.

**Figure 2:**
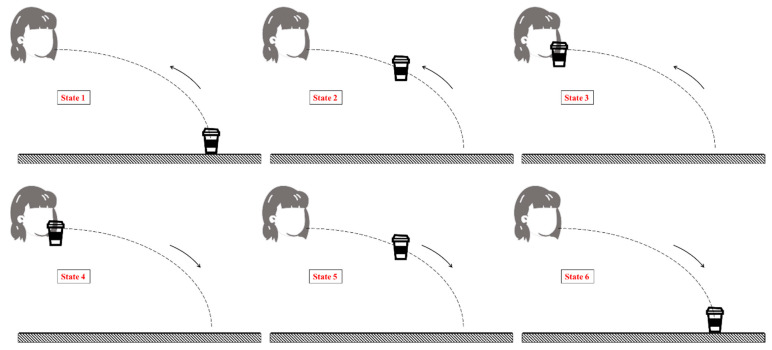
Cup position states: State 1—On the table, start position; State 2—Mid-duration between States 2 and 3; State 3—Near the mouth, before drinking; State 4—Near the mouth, after drinking, start of returning the cup to the table movement; State 5—Mid-duration between States 4 and 6; State 6—Contact with the table, end of returning movement.

The task was characterized and divided into six states describing the different positions of the cup during the task. This task, which addressed the effect of the tremor on the cup acceleration, was performed using a specially built, customized measurement system, which included a triaxial accelerometer (ADXL335 (Module by SparkFun, Niwot, CO, USA, Sensor by Analog Devices, Wilmington, MA, USA) with a range of ±3 g) attached to the cup. Data were sampled at 100 Hz using an acquisition component (NI-6008) and a computer. The cup system included an 80% volume-filled cup of water covered by a lid. The system was previously described by Roth and Rosenblum in their study [35]. The axes system used with the accelerometer is shown in Figure 1, whereas the cup position states appear in Figure 2.

To evaluate each participant’s ability to perform the drinking task, two performance measures were developed. The first measure, which is hereby termed T46, was defined as the duration between States 4 and 6. The recorded videos showed that ‘reaching the mouth’ movements, i.e., going from State 1 to State 4, were performed differently by each participant. For example, some participants held the cup near their mouth for several seconds, whereas other participants started the ‘return to the table’ movement immediately after reaching State 3. In addition, some participants tilted the cup or vertically raised the cup to mouth level at the beginning of the movement and then began moving the cup toward their mouth. Therefore, to ensure consistency among participants when analyzing the performance characteristics, we decided to consider only the time between States 4 and 6.

**Figure 3:**
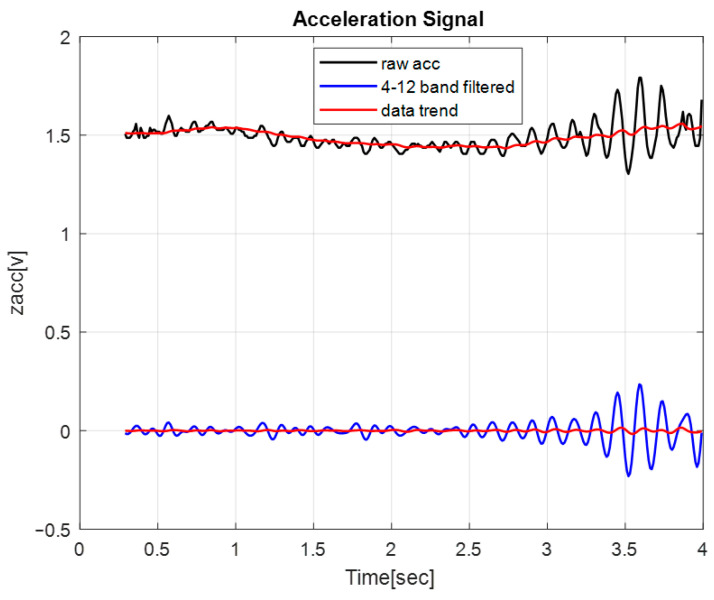
MSE Calculation.

The second measure aimed to evaluate the overall “error” caused by the tremor. To calculate this measure, first, we filtered out the signals that are associated with ET (4–12 Hz). Then, the mean-squared error (MSE) was calculated with reference to constant zero. This procedure was applied to each axis (X, Y, and Z). In addition, the overall MSE, denoted by MSE-T and constitutes the sum of the MSE components, was also calculated. Figure 3 illustrates the calculation steps for the X-axis. The black curve shows the voltage as recorded by the sensor. The filtered data are represented by the blue curve, whereas the red curves represent the data trend (by applying a moving average window).

As shown in Figure 3, as expected, the filtered data trend is approximately zero. This observation suggests that the filtered data provide a good approximation of the tremor effect. It should be noted that according to the limitations of the experimental setup regarding the acceleration measurement without a gyro element, as reported in [35], we decided to analyze and report the MSE values only for States 5 to 6.

## 3. Statistical Analysis

Statistical analysis of the demographic data was conducted using the SPSS software, Ver 25, frequency and descriptive tools. Prevalence was calculated for CADET tasks scored one or more by participants and for the tasks they noted as bothering them most. The internal reliability of the five extra digital tasks was calculated using Cronbach’s alpha. Nonparametric correlates using the Spearman test were calculated among CADET mean scores, age, and duration since noticing the tremor.

Analysis of modification data included modification prevalence in each task (for scored tasks) and modification methods. The percentages of participants who reported performing at least one task slower than had been the case prior to ET presenting, avoiding a task, and (although not asked) embarrassment and feelings of stress were also calculated.

For the objective performance-based measures, nonparametric correlates using the Spearman test were calculated among CADET mean scores and each objective performance-based measure.

## 4. Results

### 4.1. Demographics

The participants’ demographic details were described in the methods section. The participants’ ages ranged from 23 to 81 years (M=64.7, SD=15.7). Males comprised half of the sample (n=10) and 75% of the participants (n=15) had a family history of tremors based on their self-reports. Their education ranged from 12 to 27 years (M=17.2, SD=3.4) (see [37] for further details).

### 4.2. CADET Scores and Item’s Frequency Distribution, Including the Added Tasks

The internal reliability of the five extra digital-operation tasks was α=0.58, and significant correlations were found between their mean scores and the original 31 tasks on the CADET (r=0.61, p<0.01). No significant correlations, however, were found between mean scores (either of the 31 CADET tasks or the five extra tasks) and age or tremor duration.

The 31 CADET task scores ranged from 5.77 to 66.67 $\left(M=32.29,\;SD=17.43\right)$ (previously also described in [37]). The extra digital task scores ranged from 0 to 80.00 $\left(M=32.50,\;SD=20.74\right)$. Most (85%) participants reported avoiding or preferring someone else to perform at least one task.

Table 1 details the results for the CADET and extra tasks in which more than 50% of participants scored one or higher (indicating disability, need for modifications, or changed efficiency). The table also shows tasks in which 100% of participants scored zero and tasks more than 25% avoided. Regarding digital tasks not displayed (i.e., <50%), 45.0% of participants scored one or higher for the task “Select an object with the computer mouse”, and 30.0% for “Connect to digital accessories and choose an object on a mobile phone screen with your finger”.

It is important to note that 65% of participants reported other tasks that bothered them the most, ones that were not included in the CADET questionnaire. Such tasks were preparing food, eating (not just in a restaurant but also when using a fork at home), and performing work or hobby tasks, such as painting. Tasks reported with a prevalence of 35% or greater were eating (55.0%), carrying or drinking from a cup (50.0%), and writing (35.0%).

### 4.3. Reported Modifications

All participants reported implementing at least one modification, and 65.0% reported performing at least one task slower because of the tremor. These modifications are listed below. Table 2 details the tasks for which at least 50% of participants made modifications. The highest percentage of modifications were made to carrying a cup.

Participants reported various modifications to perform different daily tasks:

- Performing the task with the other hand.
- Holding the object with two hands/supporting one hand with the other.
- Changing their hand posture.
- Changing their grip force.
- Leaning their body (e.g., forearm) against the table/another surface.
- Holding the object in a different place.
- Utilizing an alternative instrument (e.g., using a spoon to eat a salad).
- Using an assistive device.
- Altering system settings (e.g., computer mouse sensitivity).

Table 3 shows the prevalence of participants making modifications in at least one task. Stabilizing one hand with the other or holding an object with two hands was the most common modification type. Although the questionnaire did not specify this task (i.e., it specified only eating in a restaurant), participants reported making modifications in eating (including at home), such as using a spoon in place of a fork (45.0%) or drinking soup instead of using a spoon. Some participants mentioned trying to relax or change eye focus or head tilt to suppress the tremor effect. The writing and carrying-a-cup tasks were among those with the highest prevalence having scores of one or more, and results show the most prevalent modification type for these tasks was holding the object with two hands/supporting one hand with the other hand (33.3% while writing and 50% while carrying a cup).

### 4.4. Additional Characteristics Reported

Although the study instruments did not directly ask about other affected body parts, stress, or embarrassment, some participants added information regarding these issues. For instance, 25% reported head tremors and 15.0% said physical activity increased the tremor. Furthermore, 65% mentioned that excitement, stress, the presence of other people (not just strangers), or the anticipation of experiencing tremors when meeting people might enhance the tremor. Overall, 55.0% reported that tremors caused embarrassment or discomfort in the presence of other people. Of these, 45.5% brought up embarrassment or discomfort during the CADET interview relative to the “eating in a restaurant” task.

### 4.5. Objective Measures of the Cup Drinking Task

**Figure 4:**
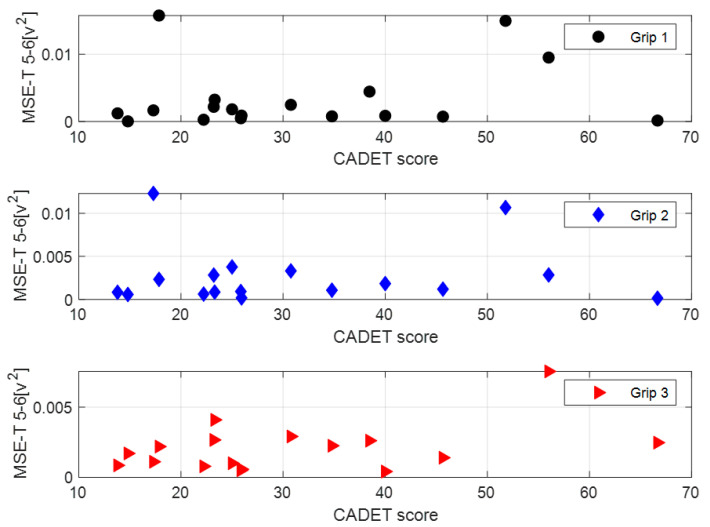
MSE-T 5–6 Measure vs. CADET Score.

In this study, 18 of the 20 participants reported difficulty in performing drinking-related tasks (Task 5 “Drinking from a glass” and/or Task 7 “Carrying a cup” of coffee in the CADET questionnaire). Figure 4 shows the MSE-T measure vs. the participants’ overall CADET score among these participants. Each of the figure’s subplots addresses a different grip type. Based on the results shown in Figure 4, no correlation is found between the CADET score and the MSE-T measure. This result was evident for all performance measures.

### 4.6. Personal Profiles

To further explore this result, we focused on four participants between the ages of 68 and 70. These participants were selected based on their reported difficulties in performing drinking-related tasks (5 and/or 7) on the CADET questionnaire. Among them, two participants, A (68 years old) and B (69 years old), had similar total CADET scores and reported difficulties in performing drinking-related tasks. Participant C (68 years old), who had the lowest total CADET score, reported no difficulties in performing drinking tasks. Finally, participant D (69 years old), who reported difficulties in drinking-related tasks, had the highest total CADET score. Table 4 outlines the obtained performance outcome measures of these four participants. We also added, from the CADET questionnaire, their reported scores for Tasks 5 and 7, as well as their overall CADET scores. It should be noted that a lower CADET score represents a lower level of reported difficulties.

Results of the comparison between the CADET-related columns of Participant A with those of Participant B suggest that these two participants should have similar objective measurement values. The difference between their objective performance measures, however, suggests that they perform the drinking task differently. On the other hand, for participants C and D, the difference is evident in both the CADET-related and the performance-related columns. These results were also verified by watching the recorded videos of these participants.

## 5. Discussion

This study focuses on characterizing daily functional difficulties and modifications that people with ET use, based on their self-reports. The CADET mean scores were similar to those of the community cases presented in [10]. Other studies showed that over time, tremor amplitude and frequency change and, from the patient’s perspective, worsen [5,39]. In our study, scores were collected at only one time point, and we found no significant correlation between self-reported disability and the duration of the tremor since it was first noticed.

As in previous works that used the Bain and Findley ADL scale [6] and CADET questionnaire [10], we found writing, carrying/holding a cup, using a spoon, and pouring among the six tasks with the highest percentages of participants reporting difficulty (or modification or changed efficiency). Participants also reported difficulty with digital equipment operation tasks, such as taking photos with a mobile phone. The five digital operation tasks included in the current study involved gross as well as fine motor tasks and postural/static, as well as movement/dynamic tasks (e.g., taking a photo or connecting a cable/plug to a digital accessory). The task of taking photos with a mobile phone is usually performed while trying to stabilize the hand/s, holding the mobile phone in the air in a desired posture against gravity. In contrast to this task, the other four tasks usually involve stabilizing the hands or the objects on the table/body. Among the five extra tasks, the “Taking a Photo” task was the most prevalent task with which participants reported having difficulty, needing to implement modifications, or losing efficiency. Thus, because digital equipment has become an inseparable tool in people’s daily functioning, results indicating that people with ET have difficulty maintaining stability when attempting to use the equipment should be considered in evaluation and intervention processes. Further, such difficulties might be considered when developing embedded digital solutions, enabling people with ET the essential daily use of these instruments.

The diverse tasks in which participants scored one or more indicate that tremor does not manifest equally across the tasks addressed in the questionnaire. These results align with previous studies that used performance-based measures and showed that tremor amplitude differed among tasks [27,40]. When listing tasks in which the tremor bothered them most, participants did not always choose those included in the questionnaire. This suggests that although, for example, drinking and writing are essential, these are not necessarily the tasks that most bother people with ET. Because the prevalence of ET rises in people over the age of 65, the daily activities of people with ET may include more hobbies and home-based activities than in younger groups. Thus, in line with the personalized medicine trend, it is important to incorporate the patients’ perspectives on their daily coping with ET [39] and consider widening the scope of self-report questionnaires with tasks relevant to patients’ perspectives, such as hobby-related activities.

Task-performance modification may be a way for people with functional deficiencies to compensate for their difficulties and optimize the performance of the task [24,25,41]. In this study, the most commonly reported physical modification was holding the object with two hands/supporting one hand with the other hand. Although not asked this question about each task, the participants’ self-reports indicated that not all tried to perform the task differently or implement different modification types. Some participants seemed unaware of the various modification types they could try for each task or did not remember which modifications they had implemented due to the tremor. Thus, participants may have implemented modifications beyond those they were able to report via the questionnaire. In addition, it seems that in some cases, participants did not feel comfortable implementing modifications in public. For example, 16 participants reported implementing modifications when carrying a cup (a more private, home-based task), whereas only six participants reported modifications when eating in a restaurant (more public). At the same time, it is important to point out that not all modification types were relevant for all tasks. For example, leaning on the table would not be a relevant modification for the carrying-a-tray task.

A better understanding and mapping of the task’s related modifications and their effect on daily activities may help characterize future support guides and lists of possible modifications that occupational therapists offer people with ET [42]. Analyzing these modifications may also help in developing mechanical tremor-suppression devices.

Although not part of the data planned to be gathered during the interviews, about half of the participants addressed the issue of discomfort or embarrassment relevant to daily functions, such as carrying a cup or eating in a restaurant. Some described how just the notion of other people’s presence enhances their tremors. Thus, we recommend that therapists relate not only to the tremor’s impact on the task but also to the task-performance environment and whether the task is performed in front of other people.

Self-report disability questionnaires are an important tool as they complement the clinical and severity tests performed by neurologists. Nevertheless, this tool may not provide detailed information per specific task when addressing multiple ADL tasks. The drinking task was chosen for the primary objective measures as this is an ADL task comprising multiple phases with different kinematic features and reported difficulty by people with ET. Results from the objective performance-based measurements of the drinking task from the presented work show that the total score of the questionnaire provides a general view of the disability in ADL rather than specific task characteristics. This was emphasized in the analysis of the results shown in Table 4. Hence, when creating an individual profile, it is important to combine objective measurements with the responses on (subjective) self-report questionnaires.

This study aimed to map activity performance features of people who are diagnosed with ET but who may not be followed routinely or medically. Moreover, it combined a self-report disability questionnaire and performance-based quantitative measures in a common ADL task of drinking to create an individual profile of each study participant.

This work had two main limitations. First, the limited sample size can be seen as a shortcoming. We were unable to recruit a larger group because of the difficulty finding participants who met the inclusion and exclusion criteria when advertising to the general population, as most people with ET do not seek medical diagnosis or treatment [1,2]. Second, 25% of participants reported head tremors, which may have affected the disability and modification types reported. In addition, as this percentage relies on reported tremors, it could be that a larger percentage of participants had head tremors.

## 6. Conclusions

This study’s results enable the mapping of individuals’ daily function capabilities from their perspective. We recommend including digital equipment operation, as well as more hobby-related and home-based tasks, in ET disability self-report questionnaires. Further, we suggest that therapists should raise patients’ awareness of possible modifications to improve task performance and thereby improve the patient’s quality of life. Furthermore, it is suggested to combine specific performance-based tests using measurement systems to design tailor-made solutions for patient-based healthcare. Finally, to provide a general understanding of the difficulties faced by people with ET when performing ADL tasks, it is recommended that future research include performance-based analyses concerning various tasks.

## Figures and Tables

**Table 1:** Task Prevalence.

Item #	Description	Prevalence
**CADET Questionnaire ^a^**		
2	Writing a letter, postcard, thank you card, or cheque ^b^	100.0%
26	Threading a needle ^b^	92.9%
7	Carrying a cup of coffee	90.0%
8	Using a spoon to drink soup	90.0%
6	Pouring milk or juice from a bottle	80.0%
10	Eating in a restaurant	75.0%
5	Drinking from a glass	70.0%
1	Signing your name	60.0%
24	Reading a book, magazine, or newspaper	60.0%
30	Tying your necktie (males) or putting on your lipstick (females) ^b^	58.3%
31	Shaving (males) or putting on your eyeliner (females) ^b^	57.1%
25	Unlocking door with a key	55.0%
9	Carrying a tray of food ^b^	52.6%
**Five extra digital equipment operation tasks ^a^**		
4	Taking a photo or video with a mobile phone	80.0%
3	Typing digits or texting messages with fingers on a mobile phone	50.0%
**Minimally difficult tasks ^c^**		
21 (CADET)	Putting on your watch	
**Avoided tasks ^d^**		
4 (five extra digital tasks)	Taking a photo or video with a mobile phone	40.0%
2 (CADET)	Writing a letter, postcard, thank you card, or cheque	31.6%

Note. CADET = Columbia University Assessment of Disability in Essential Tremor [22]. ^a^ Tasks for which at least 50% of participants scored one or more (have difficulty, need modification, loss of efficiency); ^b^ N=20, but percentages are of participants reporting on specific tasks: n=19 for Items 9 and 2 (CADET), n=12 for Items 30 and 21, and n=14 for Items 26 and 31 (spelling as in original questionnaire); ^c^ 100% of participants scored zero (no difficulty, need for modification, or loss of efficiency); ^d^ tasks at least 25% of participants reported they avoided or preferred others to perform it for them.

**Table 2:** Tasks for Which at Least 50% of Participants Reported Using Modifications.

Item #	Description	Prevalence (% Participants Using Modification)
CADET		
7	Carrying a cup of coffee	80.0
6	Pouring milk or juice from a bottle	65.0
2	Writing a letter, postcard, thank you card, or cheque	63.2
5	Drinking from a glass	60.0
24	Reading a book, magazine, or newspaper	55.0
8	Using a spoon to drink soup	50.0
Five digital tasks		
4	Taking a photo or video with a mobile phone ^a^	50.0

Note. *N* = 20. CADET = Columbia University Assessment of Disability in Essential Tremor [22]. ^a^ Included taking a picture or video with regular camera modifications.

**Table 3:** Modification Types Used in at Least One Task (N=20).

Modification Type	Prevalence (% Participants Using Modification in at Least One Task)
Holding the object with two hands/supporting one hand with the other	85.0
Leaning their body against the table/another surface	65.0
Changing the grip force	45.0
Changing their hand posture	40.0
Holding the object in a different place	35.0
Performing the task with the other hand	30.0
Utilizing an alternative instrument	30.0
Using an assistive device	30.0
Altering system settings	30.0

**Table 4:** Performance Measures and CADET.

Subject	Grip Type	T46 (s)	MSE-X (10^−6^)	MSE-Y (10^−6^)	MSE-Z (10^−6^)	MSE-T (10^−6^)	CADET Specific Task		CADET Total Score
							Drinking from a Glass—Task 5	Carrying a Cup—Task 7	
A	i	1.82	11,500	2440	1790	15,800	0	1	17.85
A	ii	1.75	536	1030	759	2320	0	1	17.85
A	iii	1.94	692	657	838	2190	0	1	17.85
B	i	1.87	92	14	144	250	0	1	22.22
B	ii	3.62	58	138	420	616	0	1	22.22
B	iii	3.1	181	122	476	780	0	1	22.22
C	i	3.9	85	128	40	253	0	0	5.77
C	ii	4.7	70	36	23	129	0	0	5.77
C	iii	3.8	39	47	21	107	0	0	5.77
D	i	1.68	2520	6370	627	9510	2	2	56
D	ii	1.31	1280	811	746	2840	2	2	56
D	iii	1.45	3500	355	3690	7450	2	2	56

## Data Availability

Data is unavailable due to privacy or ethical restrictions.

## Abbreviations

ADL	Activities of daily living
CADET	Columbia University Assessment of Disability in Essential Tremor
ET	Essential tremor
